# P30 Evaluating vancomycin continuous infusion protocol compliance and gauging therapeutic target attainment in neonates

**DOI:** 10.1093/jacamr/dlad066.034

**Published:** 2023-06-14

**Authors:** Francesca Lewis, Robert Oakley, Jennifer Hatch, Zoë Vander Elst, Anne Smits, Donovan Duffy

**Affiliations:** St George’s University Hospitals NHS Foundation Trust, London, UK; St George’s University Hospitals NHS Foundation Trust, London, UK; St George’s, University of London, London, UK; St George’s University Hospitals NHS Foundation Trust, London, UK; University Hospitals Leuven, Leuven, Belgium; Katholieke Universiteit Leuven, Leuven, Belgium; University Hospitals Leuven, Leuven, Belgium; Katholieke Universiteit Leuven, Leuven, Belgium; St George’s University Hospitals NHS Foundation Trust, London, UK

## Abstract

**Background:**

Vancomycin is commonly indicated to treat neonatal late-onset sepsis. At St. George’s Hospital, neonates with central-line access are administered vancomycin by continuous infusion. A protocol instructs a 1-hour 15 mg/kg-loading dose, followed by a creatinine clearance- (CLCR)/weight–based maintenance dose. Due to vancomycin’s narrow therapeutic margin, daily therapeutic drug monitoring (TDM) of serum concentrations is necessary to obtain safe/effective therapy. Concentrations between 15 and 25 *μ*g/mL act as a surrogate therapeutic target extrapolated from adult data.^1^

**Methods:**

A locally approved service evaluation retrospectively analysed 78 courses of vancomycin from 46 patient’s electronic prescribing and medicine administration (ePMA) records (July 2021–March 2022). Data on vancomycin administration/TDM were analysed using descriptive statistics.

**Results:**

Individual loading and maintenance doses were calculated correctly in 83% (65/78) and 97% (75/77) of records. 72% (33/46) of patients received both correctly calculated loading/maintenance doses. A gap greater than 1 hour between loading and maintenance doses was recorded in 32% (24/75) of courses. 61% (41/67) of courses had appropriately taken serum concentrations within 22–26 hours of treatment initiation. In correctly dosed patients, therapeutic vancomycin courses increased from 43% (23/54) to 53% (19/36) and 55% (17/31) between 24–48–72-hour timepoints. In pre-term neonates < 28 weeks gestational age (GA), subtherapeutic courses were consistent between 38% (15/40)–42% (11/26)–36% (8/22) at 24–48–72-hour timepoints. Supra-therapeutic courses decreased from 20% (8/40)–0% (0/26)–5% (1/22), respectively. In term neonates > 37 weeks GA, subtherapeutic courses increased from 33% (3/9)–43% (3/7)–50% (3/6) between 24–48–72-hour timepoints. As did respective supra-therapeutic courses from 11% (1/9)–14% (1/7)–17% (1/6). Insufficient course data (5) were available for neonates between 28–32/32–37 weeks GA.

**Conclusions:**

Non-therapeutic treatment worsened over 72 hours for neonates > 37 weeks GA, whereas toxic concentrations decreased over time despite consistent subtherapeutic concentrations in neonates < 28 weeks. Neonatal pharmacokinetic (PK) variability due to evolving renal function/distribution volume/non-blood protein-bound vancomycin concentrations, likely contributed to non-therapeutic concentrations. As did limitations of sample size/non-capture of dosing amendment impact. Population PK–derived dosing based on covariates explaining vancomycin PK variability could be used to improve neonatal target attainment. Whereas, neonatal area under the vancomycin concentration time curve targets supported by clinical data are needed to benchmark the therapeutic target. This will provide insight into if lower concentrations adequately treat common infections with coagulase-negative organisms.^1^ A quality improvement project addressing protocol ePMA interface issues and implementing a dose-amendment nomogram may improve dose calculation/monitoring. Delays between manually input loading/maintenance dose administration times onto the ePMA system require investigation before dosing revisions. Aspects of protocol compliance require improvement. Therapeutic target attainment was erratic, but reflective of the literature.^1^ Optimized dosing is required for specific neonatal subgroups.
